# Crouch Gait in Dravet Syndrome

**DOI:** 10.15844/pedneurbriefs-30-11-2

**Published:** 2016-11

**Authors:** Laura Black, Deborah Gaebler-Spira

**Affiliations:** 1 Department of Physical Medicine and Rehabilitation, Northwestern University Feinberg School of Medicine, Chicago, IL

**Keywords:** Dravet Syndrome, Neurologic Gait Disorders, Nav1.1 Voltage Gated Sodium Channel

## Abstract

Investigators from Necker Enfants Malades Hospital, Sorbonne Paris Cite University, Raymond Poincare University, and Paris Descartes University studied motor neuron function in children with Dravet syndrome (DS).

Investigators from Necker Enfants Malades Hospital, Sorbonne Paris Cite University, Raymond Poincare University, and Paris Descartes University studied motor neuron function in children with Dravet syndrome (DS). DS is a rare condition that involves mutation of the SC1NA gene, which encodes a voltage-gated sodium channel isoform known as Nav1.1. *SCN1A* is expressed in neuronal cell membranes throughout the central and peripheral nervous systems. Children with DS generally present with seizures in the first year of life, and often develop ataxia, crouch gait, and other orthopedic abnormalities. The study authors performed a neurologic examination of twelve children, ages 2 to 17, with a confirmed diagnosis of DS and mutation of the *SCN1A* gene. Nerve conduction studies (NCS) of the peroneal, tibial, and median motor nerves, and the median and sural sensory nerves and electromyography (EMG) of two distal lower limb muscles and one upper limb muscle were conducted on each child. All children in the study showed gait disturbances and delays in fine motor skills: sixty percent of the children began walking after 18 months of age. The subjects over age six showed crouch gait and joint deformities. Seven of the ten children who tolerated a complete EMG and NCS study showed motor unit potentials (MUAPs) consistent with a motor neuropathy, and three had some MUAP characteristics consistent with a motor neuropathy. Two children had normal EMG and NCS studies. The authors concluded that motor neuron dysfunction due to the *SCN1A* genetic abnormality may be a potential etiology in gait dysfunction in children with DS. [1]

COMMENTARY. Parents and neurologists are increasingly concerned about functional declines, especially crouched walking, in the children they follow with DS. A previous article by the Australian group Rodda, et al., documented the characteristics by age in a small sample of children but did not explore etiology [2]. The neurologists begin this exploration by examining the peripheral nervous system which makes sense given the absence of pyramidal signs and focal defects, and decreased reflexes. Crouching begins at about the age of 6 years old, when the trajectory of decline begins. Given that electromyography can be technically difficult in children under five years, the initial phase of gait disturbance in DS still remains unclear. Though a small sample, this inquiry begins to help us understand crouch gait in the population of children with DS. Crouched gait is well known to decrease endurance as it decreases quadriceps strength, increases fatigue, and causes lever arm dysfunction. Attention to gait and mobility is important since it allows for participation in the International Classification of Functioning, Disability, and Health (ICF). Collaboration with rehabilitation professionals ensures attention to the ICF paradigm and maximizes each child’s social participation [3].
